# Health-promoting behaviors and social support of women of reproductive age, and strategies for advancing their health: Protocol for a mixed methods study

**DOI:** 10.1186/1471-2458-11-191

**Published:** 2011-03-28

**Authors:** Azam Baheiraei, Mojgan Mirghafourvand, Eesa Mohammadi, Saharnaz Nedjat, Sakineh Mohammad-Alizadeh Charandabi, Fatemeh Rajabi, Reza Majdzadeh

**Affiliations:** 1 Department of Reproductive Health, Tehran University of Medical Sciences, Tehran, Iran; 2 Center for Community-Based Participatory Research, Tehran University of Medical Sciences, Tehran, Iran; 3 Department of Nursing, Tarbiat Modares University, Tehran, Iran; 4 Department of Epidemiology and Biostatistics, Tehran University of Medical Sciences, Tehran, Iran; 5 Midwifery Department, Tabriz University of Medical Sciences, Tabriz, Iran; 6 School of Public Health and Institute of Public Health Research, Tehran University of Medical Sciences, Tehran, Iran

## Abstract

**Background:**

Determining the health-promoting behaviors of women during the important period of reproduction provides valuable information for designing appropriate intervention programs for advancing women's health. There is no study on the health-promoting behaviors of women of reproductive age in Iran. Thus, the aim of this study is to explore these health-promoting behaviors for the purpose of developing comprehensive and culturally sensitive health advancement strategies for Iranian women.

**Methods/Design:**

This study has a sequential explanatory mixed methods design. The follow-up explanation model is used to elaborate the quantitative results by collecting qualitative data from participants who could best assist in elucidating the results. The study is conducted in two sequential phases. The first phase is a population-based cross-sectional survey in which 1350 Iranian women of reproductive age are selected by proportional random multistage cluster sampling of the 22 main municipal sectors of Tehran, Iran. Questionnaires are completed through a face-to-face interview. The second phase is a qualitative study in which participants are selected using purposive sampling in the form of extreme case sampling on the basis of health-promoting behavior scores. The qualitative phase is based on data collected from focus group discussions or individual in-depth interviews. A conventional qualitative content analysis approach is used, and the data are managed with a computer-assisted program. Women's health-promoting strategies are developed using the qualitative and quantitative results, a review of the related literature, and the nominal group technique among experts.

**Discussion:**

The findings of this mixed methods sequential explanatory study, obtained using a culturally sensitive approach, provide insights into the health behavioral factors that need to be considered if preventive strategies and intervention programs are to be designed to promote women's health in the community.

## Background

Health-promoting behaviors are among the main determinants of health that have been recognized as underlying factors in disease prevention [1]. Modifying lifestyle factors could potentially prevent many cases of heart disease and types 2 diabetes [2]. Thus, health-promoting behaviors and a healthy lifestyle should be considered as major strategies to improve and maintain health [3]. Promoting women's health is necessary during the reproductive years, the period when health issues such as pregnancy-related diseases and breastfeeding emerge. Women's health also influences the health status of other family members, including those of children.

Health-promoting behaviors in women have shown a discrepancy from country to country because of sociocultural determinants. The inconsistent results on health-promoting behavior status observed in different populations can be due to the effects of a range of personal, social, economic, and environmental factors that determine an individual's health condition. Health behaviors are influenced by social norms, culture, mass media, national health policies, advertising practices, and physical and social environments [4].

Social support affects health-promoting behaviors. The members of a social support network who are sources of positive and negative feelings may have detrimental physiological consequences on health [5]. Social support has been viewed as integral to health promotion because of its assistance in reaching an individual's physical and emotional needs, as well as buffering the effects of stressful events on the quality of life [6]. According to Pender (1996), social support is identified as "a subjective feeling of belonging, being loved, esteemed, valued, and needed for oneself, not for what one can do for others" [7].

In Iran, the population in 2006 was 70495789, comprising 34629420 females of which about 60 percent were of reproductive age (15-49 years) [8]. Nevertheless, only one quantitative study identified health-promoting behaviors and their relation with perceived religious support in elderly women [9], and no qualitative or quantitative study has been conducted on this topic among women of reproductive age. With a better understanding of the health behaviors of women of reproductive age, and their association with social support and sociodemographic characteristics, it is possible to promote the health of this social group in different aspects in order to improve their quality of life.

This study is designed to assess the various aspects of the health behaviors of women of reproductive age and to provide basic information for the national and provincial authorities and policy makers toward the appropriate planning and allocation of resources based on priorities that in turn help to promote women's health. Most of the researches about health-promoting behaviors have been conducted with a quantitative approach, and limited qualitative data are available on women's experience of health-promoting behaviors and social support. Moreover, none of the studies have used the mixed methods approach to gain a better understanding of health-promoting behaviors and their relation to social support in women of reproductive age for the purpose of developing strategies for advancing women's health.

### The study aims

The aim of this mixed methods study is to determine health promoting behaviors as well as their determinants including perceived social support and sociodemographic characteristics. Furthermore, women's experience of health promoting behaviors will be explored. On the basis of the findings, comprehensive and culturally sensitive strategies for promoting health behaviors of Iranian women will be developed.

The specific objectives are:

1. To determine the health-promoting behaviors

2. To determine the perceived social support

3. To determine the association between the health-promoting behaviors and perceived social support and the sociodemographic characteristics

4. To explore the women's experience of health-promoting behaviors

5. To offer health-promoting strategies for Iranian women of reproductive age

## Methods/Design

**Figure 1:**
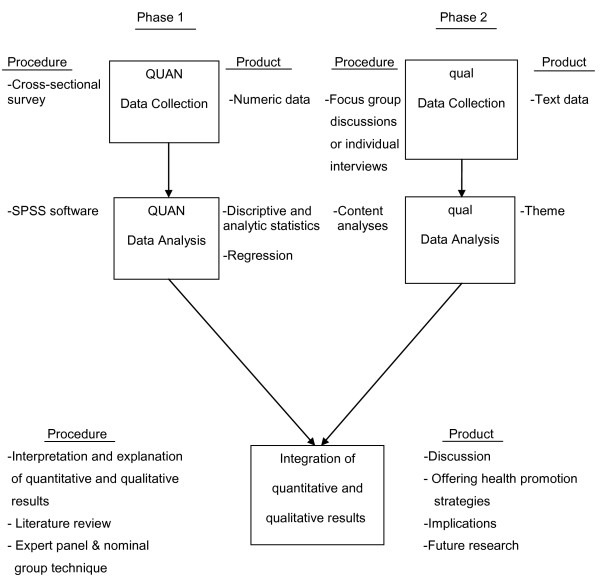
**Study visual diagram**. The file includes an overview of the study design.

The present study was designed as a mixed methods sequential explanatory approach valuing both objective and subjective information. The follow-up explanation model is used to explain the quantitative results. The explanatory sequential approach is a two-phase mixed methods design that begins with the collection and analysis of quantitative data, followed by the collection and analysis of qualitative data to explain and enrich the quantitative findings [10,11]. In this model, the researcher identifies specific quantitative results that need additional explanation, such as statistical differences among groups, individuals who scored at extreme levels, or unexpected findings. Then, the researcher collects qualitative data from those participants who can better help explain these findings [12]. The questions used in the qualitative phase are informed by the findings from the quantitative data. The quantitative data are collected first and given priority in this study. The integration of results occurs in the interpretation and explanation of the quantitative and qualitative results (Figure 1).

### Phase one: quantitative study

This phase is designed as a population-based cross-sectional study to assess health-promoting behaviors and their relations with perceived social support and sociodemographic characteristics in a representative sample of Iranian women of reproductive age. Extreme scores are detected at this stage. The source population is every woman of reproductive age (15-49 years) living in Tehran.

### Sample size and sampling method

The sample size calculation is based on the largest standard deviation of the subscales in

previous studies that have been 7.49 [7,9], with type 1 error (alpha) of 5% and accuracy of 0.5; approximately 900 samples are calculated, which, due to a probable design effect of 1.5 (900 × 1.5), reached a sample size of 1350 people. Proportional random multistage cluster sampling is done in the 22 main municipal sectors of Tehran, the capital of Iran. The blocks in each zone are chosen randomly and weighted using the number of individuals in that block. Systematic sampling is conducted in each block. The distance between households is one-tenth of the number of households in the block. Thus, ten households are chosen from each block, and a woman of reproductive age is chosen from these households.

### Eligibility criteria

1. Iranian nationality

2. Able to speak

3. Residing in Tehran and within the 15-49-year age group

4. Neither pregnant nor in the puerperium period

5. No diagnosed severe mental disorders that make her unable to respond to the questions

### Scales and data collection

The questionnaire comprising three sections was completed through a face-to-face interview. It consists of questions on sociodemographic characteristics, the Health-Promoting Lifestyle Profile II (HPLP-II), and the Personal Resource Questionnaire 85 Part 2 (PRQ85-PART2).

The HPLP-II questionnaire was developed by Walker and colleagues (1987) based on Pender's health promotion model to measure health-promoting behaviors. This questionnaire consists of 52 items on the six aspects of health-promoting behaviors, including nutrition, physical activity, spiritual growth, health responsibility, stress management, and interpersonal relations. The health promotion model represents a theoretical viewpoint that explores the factors contributing to health-promoting behaviors and the improvement of health and quality of life [13]. The PRQ-85 questionnaire was designed by Brandt and Weinert (1987) to measure the perceived social support. The PRQ-85-Part 2 is a 25-item scale based on the five dimensions of support including worth, social integration, intimacy, nurturance, and assistance [14,15]. The questionnaires were translated from English into Persian, and their validity and reliability have been examined in previous studies in different populations in Iran [9,16]. Both questionnaires are available as supplementary files (Additional File 1).

In the present study, in order to examine their content validity, the questionnaires are given to 5 experts on the subject, an expert in methodology, and 3 non-professional women of reproductive age in the society. After collecting the opinions of these experts, the content validity index is calculated and possible modifications are made. To assess the face validity and reliability regarding repeatability and internal consistency, a test-retest is performed on 20 women, and the intra-class correlation coefficient and Cronbach alpha values are calculated. The cut-off point for both calculations is 0.70.

### Data analysis

The analyses are performed using the SPSS software. Basic descriptive statistics, including the mean, standard deviation, and frequency, are calculated. Statistical tests such as the independent t-test and the Pearson test are used to estimate correlations. Multivariable linear regression analysis will be used to predict the impact of each of the independent variables (sociodemographic variables and social support) on the dependent variable (health promoting behaviour and its subscales) and determine the variance.

### Phase two: qualitative study

#### Sampling method

Purposive sampling is conducted. To determine the index for extreme cases, the range of health promotion scores is considered. Women who scored less or higher than 10% of the attainable scores are selected purposefully as extreme cases.

#### Data collection

Data are collected through focus group discussions or individual in-depth interviews, if required. The interviews are conducted in locales convenient to the women. Before conducting the interviews, the research team reviews the questions, and the ways to obtain valid data and focus on research questions. During the interviews, the data are recorded through note taking and tape-recording. Data collection is continued until saturation is reached and no new themes emerge in the interviews.

#### Data analysis

The interpretation of the data is ensured with the help of participants who are randomly selected to compare their perspectives with those of the research team. The research team reviews the interviews, and extracts codes and categories to assess the accuracy of the coding process [17]. The conventional content analysis approach is used, in which themes and categories are explored to reveal the women's experiences of health-promoting behaviors and social support. The advantage of using the conventional qualitative content analysis approach is that of deriving coding categories from the raw data directly, without imposing preconceived categories or previous theoretical perspectives. The knowledge generated from the content analysis method is based on the participant's unique viewpoints and the results derived from the interviews [18]. Moreover, the constant comparison method is used during the conduct of the research. A computer-assisted program is used to manage the data.

#### Integration of quantitative and qualitative data

Health-promoting strategies for Iranian women of reproductive age are developed by using the quantitative and qualitative findings, reviewing the related literature on strategies for promoting health behaviors, and employing the nominal group technique (NGT) among experts from different disciplines to increase the variety of views on the discussed topics.

#### Ethical approval

Written informed consent is taken from each participant. The Ethics Committee of the Tehran University of Medical Sciences in Tehran, Iran approved the protocol of this study (code number: 89-02-28-10802).

## Discussion

Promoting women's health will help countries reach many of the Millennium Development Goals [19]. The present study provides information and robust data on women's health-promoting behaviors through a culturally sensitive approach. The collection of both quantitative and qualitative data allow for a better understanding of the research goals. By using qualitative and quantitative research methods, this mixed methods study enlightens the quantitative results found by a complementary study through a survey among a representative sample of Iranian women. To develop strategies for promoting health behaviors, the qualitative and quantitative results, a review of the related literature on health-promoting behaviors, and the NGT among experts are used. In comparison with other techniques such as Delphi, focus groups, and brainstorming, the NGT has a number of advantages, including the immediate dissemination of results to the group, which promotes satisfaction with participation, and the highly structured nature of the process, which minimizes researcher bias [20].

The study's findings may help public health educators, health promoters, social workers, and policy makers to understand the critical role of effective, culturally based preventive strategies as well as women's needs, particularly during the reproductive years. This study also provides some insights into the health behavior factors that need to be considered if effective strategies and intervention programs are to be designed to promote women's health and, subsequently, the health of their families in Iran and those of other women who may have similar beliefs and practices that need to be dealt with effectively.

## List of abbreviations

HPLP-II: Health-Promoting Lifestyle Profile-II; PRQ85-PART2: Personal Resource Questionnaire 85-Part 2; WHO: World Health Organization; NGT: Nominal Group Technique.

## Competing interests

The authors declare that they have no competing interests.

## Authors' contributions

All the authors contributed to the conception and design of the study. MM drafted the first version of the manuscript. AB, EM, MM, and SN revised the manuscript. AB critically reviewed the manuscript for important intellectual content. All authors approved the final version.

## Pre-publication history

The pre-publication history for this paper can be accessed here:

http://www.biomedcentral.com/1471-2458/11/191/prepub

## Supplementary Material

Additional file 1 **Questionnaires**. The file includes both initial questionnaires. Click here for file
