# Supplementary material for: Health-promoting behaviors and social support of women of reproductive age, and strategies for advancing their health: Protocol for a mixed methods study
Source: BMC Public Health. 2011 Mar 28;11:191. doi: 10.1186/1471-2458-11-191 (PMC3073903; doi:10.1186/1471-2458-11-191)
Supplement: Additional file 1 — Questionnaires. The file includes both initial questionnaires. [file 1471-2458-11-191-S1.DOC]

**Health-Promoting Lifestyle Profile II**

**DIRECTIONS: This questionnaire contains statements about your present way of life or personal habits. Please respond to each item as accurately as possible, and try not to skip any item. Indicate the frequency with which you engage in each behavior by circling:**

**N for never, S for sometimes, O for often, or R for routinely**

**1. Discuss my problems and concerns with people close to me. N S O R**

**2. Choose a diet low in fat, saturated fat, and cholesterol. N S O R**

**3. Report any unusual signs or symptoms to a physician or other health professional. N S O R**

**4. Follow a planned exercise program. N S O R**

**5. Get enough sleep. N S O R**

**6. Feel I am growing and changing in positive ways. N S O R**

**7. Praise other people easily for their achievements. N S O R**

**8. Limit use of sugars and food containing sugar (sweets). N S O R**

**9. Read or watch TV programs about improving health. N S O R**

**10. Exercise vigorously for 20 or more minutes at least three times a week (such as N S O R**

**brisk walking, bicycling, aerobic dancing, using a stair climber).**

**11. Take some time for relaxation each day. N S O R**

**12. Believe that my life has purpose. N S O R**

**13. Maintain meaningful and fulfilling relationships with others. N S O R**

**14. Eat 6-11 servings of bread, cereal, rice and pasta each day. N S O R**

**15. Question health professionals in order to understand their instructions. N S O R**

**16. Take part in light to moderate physical activity (such as sustained walking N S O R**

**30-40 minutes 5 or more times a week).**

**17. Accept those things in my life which I cannot change. N S O R**

**18. Look forward to the future. N S O R**

**19. Spend time with close friends. N S O R**

**20. Eat 2-4 servings of fruit each day. N S O R**

**21. Get a second opinion when I question my health care provider's advice. N S O R**

**22. Take part in leisure-time (recreational) physical activities (such as swimming, N S O R**

**dancing, bicycling).**

**23. Concentrate on pleasant thoughts at bedtime. N S O R**

**24. Feel content and at peace with myself. N S O R**

**25. Find it easy to show concern, love and warmth to others. N S O R**

**26. Eat 3-5 servings of vegetables each day. N S O R**

**27. Discuss my health concerns with health professionals. N S O R**

**28. Do stretching exercises at least 3 times per week. N S O R**

**29. Use specific methods to control my stress. N S O R**

**30. Work toward long-term goals in my life. N S O R**

**31. Touch and am touched by people I care about. N S O R**

**32. Eat 2-3 servings of milk, yogurt or cheese each day. N S O R**

**33. Inspect my body at least monthly for physical changes/danger signs. N S O R**

**34. Get exercise during usual daily activities (such as walking during lunch, using N S O R**

**stairs instead of elevators, parking car away from destination and walking).**

**35. Balance time between work and play. N S O R**

**36. Find each day interesting and challenging. N S O R**

**37. Find ways to meet my needs for intimacy. N S O R**

**38. Eat only 2-3 servings from the meat, poultry, fish, dried beans, eggs, and N S O R**

**nuts group each day.**

**39. Ask for information from health professionals about how to take good care N S O R**

**of myself.**

**40. Check my pulse rate when exercising. N S O R**

**41. Practice relaxation or meditation for 15-20 minutes daily. N S O R**

**42. Am aware of what is important to me in life. N S O R**

**43. Get support from a network of caring people. N S O R**

**44. Read labels to identify nutrients, fats, and sodium content in packaged food. N S O R**

**45. Attend educational programs on personal health care. N S O R**

**46. Reach my target heart rate when exercising. N S O R**

**47. Pace myself to prevent tiredness. N S O R**

**48. Feel connected with some force greater than myself. N S O R**

**49. Settle conflicts with others through discussion and compromise. N S O R**

**50. Eat breakfast. N S O R**

**51. Seek guidance or counseling when necessary. N S O R**

**52. Expose myself to new experiences and challenges. N S O R**

**Personal Resource Questionnaire 85- Part 2**

**Below are some statements with which some people agree and others disagree. Please read each statement and CIRCLE the response most appropriate for you. There is no right or wrong answer.**

**1 = STRONGLY DISAGREE**

**2 = DISAGREE**

**3 = SOMEWHAT DISAGREE**

**4 = NEUTRAL**

**5 = SOMEWHAT AGREE**

**5 = AGREE**

**5 = STRONGLY AGREE**

**STATEMENTS**

a. There is someone I feel close to who makes me feel secure.

1 2 3 4 5 6 7

b. I belong to a group in which I feel important.

1 2 3 4 5 6 7

c. People let me know that I do well at my work (job, homemaking).

1 2 3 4 5 6 7

d. I can’t count on my relatives and friends to help me with problems.

1 2 3 4 5 6 7

e. I have enough contact with the person who makes me feel special.

1 2 3 4 5 6 7

f. I spend time with others who have the same interests that I do.

1 2 3 4 5 6 7

g. There is little opportunity in my life to be giving and caring to another person.

1 2 3 4 5 6 7

h. Others let me know that they enjoy working with me (jobs, committees, projects).

1 2 3 4 5 6 7

i. There are people who are available if I needed help over an extended period of time.

1 2 3 4 5 6 7

j. There is no one to talk to about how I am feeling.

1 2 3 4 5 6 7

k. Among my group of friends we do favors for each other.

1 2 3 4 5 6 7

l. I have the opportunity to encourage others to develop their interests and skills.

1 2 3 4 5 6 7

m. My family lets me know that I am important for keeping the family running.

1 2 3 4 5 6 7

n. I have relatives or friends that will help me out even if I can’t pay them back.

1 2 3 4 5 6 7

o. When I am upset there is someone I can be with who lets me be myself.

1 2 3 4 5 6 7

p. I feel no one has the same problems as I.

1 2 3 4 5 6 7

q. I enjoy doing little “extra” things that make another person’s life more pleasant.

1 2 3 4 5 6 7

r. I know that others appreciate me as a person.

1 2 3 4 5 6 7

s. There is someone who loves and cares about me.

1 2 3 4 5 6 7

t. I have people to share social events and fun activities with.

1 2 3 4 5 6 7

u. I am responsible for helping provide for another person’s needs.

1 2 3 4 5 6 7

v. If I need advice there is someone who would assist me to work out a plan for dealing with a situation.

1 2 3 4 5 6 7

w. I have a sense of being needed by another person. 1 2 3 4 5 6 7

x. People think that I’m not as good a friend as I should be. 1 2 3 4 5 6 7

y. If I got sick, there is someone to give me advice about caring for myself. 1 2 3 4 5 6 7
